# Development and validation of a serious game for nursing leadership in Primary Health Care

**DOI:** 10.1590/0034-7167-2025-0590

**Published:** 2026-09-25

**Authors:** Caren Cristina Wiles Della Méa da Fonseca, Alacoque Lorenzini Erdmann, Carla Sílvia Fernandes, Alexandre Pazetto Balsanelli, Ana Izabel Jatobá de Souza, Keyla Cristiane do Nascimento

**Affiliations:** I Universidade Federal de Santa Catarina. Florianópolis, Santa Catarina, Brazil; II Escola Superior de Saúde, Instituto Politécnico de Viana do Castelo. Viana do Castelo, Portugal; III Universidade Federal de São Paulo. São Paulo, São Paulo, Brazil

**Keywords:** Validation Study, Educational Technology, Leadership, Nursing, Primary Health Care., Estudio de Validación, Tecnología Educacional, Liderazgo, Enfermería, Atención Primaria de Salud.

## Abstract

**Objectives:**

to develop and validate a serious game for nursing leadership among Primary Health Care nurses.

**Methods:**

a methodological, quantitative, descriptive study conducted in two stages: content validity was assessed by 13 experts via e-Delphi, and usability was assessed by 14 nurses using the System Usability Scale and the Serious Educational Game in Nursing Appraisal Scale. Data were analyzed using descriptive statistics.

**Results:**

the global Content Validity Index was 0.93. Mean usability score was 92.0; the educational strategy appraisal score was 97.9. The final version of the serious game included a board with an improved layout, 75 revised cards, and refined rules, making it an educational resource applicable to professional practice.

**Conclusions:**

the serious game proved to be a valid educational technology with high usability and was effective in supporting the development of leadership competencies among PHC nurses and may serve as an innovative alternative to traditional educational practices.

## RESUMEN

**Objetivos:**

desarrollar y validar un juego serio sobre liderazgo para enfermeros de Atención Primaria de Salud.

**Métodos:**

estudio metodológico, cuantitativo y descriptivo, en dos etapas: validación de contenido por 13 especialistas mediante e-Delphi y evaluación de la usabilidad por 14 enfermeros, con *System Usability Scale* y *Serious Educational Game in Nursing Appraisal Scale*. Los datos se analizaron mediante estadística descriptiva.

**Resultados:**

el índice de validez de contenido global fue de 0,93. La usabilidad alcanzó 92 puntos y la valoración del juego como estrategia educativa, 97,9. La versión final incluyó un tablero con diseño mejorado, 75 cartas revisadas y reglas ajustadas, y resultó aplicable a la práctica profesional.

**Conclusiones:**

el juego serio resultó ser una tecnología educativa válida, con alta usabilidad y eficaz para desarrollar competencias de liderazgo entre enfermeros de APS, y se perfila como alternativa innovadora frente a las prácticas educativas tradicionales.

## INTRODUCTION

Leadership is regarded as an essential competency in nursing practice, with a direct impact on the quality of patient care, family relationships, team coordination, and the management of health services^(1)^. Investing in leadership development and nurse preparation is a key strategy for strengthening health systems and advancing the Sustainable Development Goals (SDGs) set out in the 2030 Agenda^(2,3)^.

In Primary Health Care (PHC), this challenge is even more complex, as nurses must draw not only on technical skills but also on relational and managerial competencies required to work in dynamic, complex environments^(4)^. This process requires ongoing reflection, an ethical stance, the ability to build trusting relationships with patients and interprofessional teams, and the capacity to effectively foster collaborative work^(1)^.

Key nursing leadership competencies include decision-making, conflict management, assertive communication, critical thinking, and empathy. These competencies improve administrative, clinical, educational, and research practice and should be encouraged during training so that future nurses feel more confident and better prepared to apply them^(5)^. Recent studies, however, show that gaps in training and difficulties in consolidating these competencies persist in both initial education and continuing professional development^(5,6)^.

Evidence from PHC points to challenges involving nurses’ managerial and leadership competencies, reinforcing the need for structured continuing education programs and initiatives focused on leadership development^(7)^. Investing in educational processes aligned with the real-world demands of health services is essential to transform care practices, improve the effectiveness of health interventions, and enhance the quality of care^(6)^.

In this context, educational technologies have become established as effective alternatives for health professional education and development. Serious games, a category of educational games, stand out for promoting participation, motivation, and collaborative knowledge-building while incorporating elements of healthy competition, critical reflection, and creative engagement^(8)^. Board games, in particular, offer an active, dynamic, and innovative pedagogical resource that can turn complex content into accessible, participatory experiences, fostering meaningful learning and the practical application of knowledge^(9,10)^.

Despite the growing use of these technologies, few studies have examined educational resources focused on nursing leadership in PHC. This gap limits scientific progress and restricts the incorporation of innovative pedagogical strategies into everyday health service practice. In this regard, incorporating educational games offers a methodological alternative for developing contextualized knowledge that can be applied across different situations.

## OBJECTIVES

To develop and validate a serious game on leadership for Primary Health Care nurses.

## METHODS

### Ethical aspects

The study complied with national and international ethical standards for research involving human participants. It was approved by the Research Ethics Committee of the Federal University of Santa Catarina (UFSC) under CAAE No. 78961724.0.0000.0121 and Approval No. 6.818.857. The images used in the game were obtained from a royalty-free image bank, in accordance with Canva^®^’s copyright terms.

### Study design, period, and setting

This was a methodological, quantitative, descriptive study based on the Guidelines for reporting reliability and agreement studies (GRRAS) from the EQUATOR Network, adapted for educational technology validation studies. It was conducted between February 2024 and April 2025 in a state capital in the South Region of Brazil. The game was developed according to the Hexa-Tau model for educational board games^(11)^. The study was conducted in three interrelated stages: 1) serious game development and design, 2) expert content validity, and 3) usability and applicability assessment with PHC nurses.

### Population; inclusion and exclusion criteria

In the content validity stage, experts were selected through nonprobability convenience sampling using the Lattes Platform of the National Council for Scientific and Technological Development (CNPq), based on expertise in leadership. Inclusion criteria were being a nurse, having experience in nursing leadership in PHC, and having worked at this level of care for at least 1 year. Email invitations were sent to 32 professionals, each containing a link to an electronic form. After agreeing to participate, individuals were given access to the instrument; if they declined, no further steps were taken. Those who did not return the instrument within the established timeframe were excluded.

For the usability assessment, 29 nurses working in PHC in a municipality in South Brazil were invited. Inclusion criteria were being a PHC nurse and having worked in this role for at least 1 year. Professionals who were on vacation or leave during the data collection period were excluded. Participation in the study did not interfere with the nurses’ professional activities because data collection took place outside patient care activities. Importantly, all nurses were invited to take part in the game activities regardless of their decision about study participation, as these activities were designed to be playful and inclusive. The questionnaires were administered only to those who agreed to participate in the study, in accordance with the Informed Consent Form.

### Study protocol

#### Stage 1 - Serious game development

In the first stage, the game was designed and developed following the Hexa-Tau model^(11)^. An analog board game was selected for its accessibility, ease of replication, and potential to promote interaction during in-person educational activities. The game, titled “LíderEnf - Primary Health Care”, was designed in a rectangular format (42 × 30 cm) and consisted of 30 colored spaces, each representing different leadership skills. The board path was designed to form the letters “L” and “E”, the initials of the Portuguese words Liderança and Enfermagem, alluding to the game’s title. The spaces, in red, blue, green, yellow, and white, were arranged in a continuous sequence and connected by ladders representing strategic advances, with colors used to identify the thematic categories. The game set includes four pawns, one six-sided die, a rulebook, and 75 cards containing questions and statements organized into thematic categories related to nursing leadership competencies: effective communication, decision making, conflict management, team motivation, and informational cards (“Did you know?”)^(11)^.

#### Stage 2 - Content validity

Content validity was assessed in terms of clarity, relevance, and organization using the e-Delphi technique^(12)^. The questionnaire, created in Google Forms^®^, included expert characteristics (age, sex, educational background, and other aspects) and 32 items distributed across six dimensions: Objectivity, Content, Relevance, Organization, Layout and images, and Linguistic accuracy. Each item was rated on a five-point Likert scale (from 1 = Strongly disagree to 5 = Strongly agree)^(13)^. For items scored below 4, experts were asked to provide comments and suggestions. The process was carried out in two rounds until the minimum consensus threshold of 80% for the Content Validity Index (CVI)^(13)^ was reached.

#### Stage 3 - Usability assessment

Usability was assessed in person with nurses during six sessions held at PHC health units, scheduled according to participants’ availability. Because this was a board game, each session included at least two participants to ensure the activity could take place as intended. Sessions lasted about 1 hour and included one full round of the game followed by administration of the assessment instruments. Two instruments were used: the System Usability Scale (SUS), in its Portuguese version translated and validated for use in Brazil, consisting of 10 items^(14)^, and the Serious Educational Game in Nursing Appraisal Scale (SEGiNAS), which includes 20 statements distributed across three dimensions (Engagement and Teaching Effectiveness, Learning Impact and Practical Application and Content Relevance and Clarity)^(15)^. Both instruments used a five-point Likert scale ranging from “Strongly disagree” to “Strongly agree”.

### Data analysis and statistics

Data were organized in Microsoft Excel 365^®^ spreadsheets and analyzed using descriptive statistics, with absolute and relative frequencies used to characterize the participants. The CVI was calculated by dividing the sum of responses scored as “4” and “5” by the total number of responses for each item. A minimum value of 0.80 (80%) was considered acceptable. Items with lower values were revised according to the experts’ suggestions and reassessed in a second round, whereas those that had already reached consensus in the first round were considered valid. For the System Usability Scale, scores were calculated according to the original recommendations: for odd numbered items, 1 was subtracted from the assigned score; for even-numbered items, the assigned score was subtracted from 5. The sum of these values was multiplied by 2.5, resulting in a total score ranging from 0 to 100. Scores were classified according to mean System Usability Scale values associated with qualitative adjectives: 12.5 (Worst Imaginable), 20.3 (Awful), 35.7 (Poor), 50.9 (OK), 71.4 (Good), 85.5 (Excellent), and 90.9 (Best Imaginable)^(14)^. The SEGiNAS was analyzed using a simple summative score, with a minimum score of 80 points (80% agreement) considered satisfactory.

## RESULTS

Thirteen expert nurses with leadership experience participated in the validation process; eight (61.5%) were between 40 and 50 years old, and most were women (84.6%). The mean time since graduation was 21.6 years. Geographically, nine (69.2%) were from the South Region of Brazil, and four (30.8%) were from the Southeast Region of Brazil. In terms of academic qualifications, five (38.5%) had completed postdoctoral training, four (30.8%) held a doctoral degree, and four (30.8%) held a master’s degree. Regarding professional activity, six (46.2%) were faculty members and seven (53.8%) were PHC nurses.

In the e-Delphi process, experts assessed the content validity items individually based on agreement frequency and CVI calculation. The results from both rounds are summarized in Table 1, which presents the CVI values and mean scores for each dimension assessed.

**Table 1:** Content Validity Index and mean scores for the items assessed in the two rounds of the e-Delphi process (n = 13; n = 9), Florianópolis, Santa Catarina, Brazil, 2025

Validation dimensions	e-Delphi 1 (n = 13)		e-Delphi 2 (n = 09)	
	CVI	Mean (SD)	CVI	Mean (SD)
**Objectivity**				
Clear objectives, consistent with the theme of leadership	0.90	4.58 (0.66)	0.90	4.58 (0.66)
**Organization**				
The game’s organization and rules are clear, objective, consistent, and easy to understand	0.87	4.28 (0.91)	0.88	4.36 (0.83)
**Content**				
Effective communication - Relevant, appropriate content presented clearly and objectively	0.79	4.08 (1.03)	0.98	4.57 (0.57)
Decision-making - Relevant, appropriate content presented clearly and objectively	0.83	4.19 (1.00)	0.91	4.34 (0.84)
Conflict management - Relevant, appropriate content presented clearly and objectively	0.78	4.10 (1.08)	0.97	4.50 (0.62)
Team motivation - Relevant, appropriate content presented clearly and objectively	0.84	4.10 (1.02)	0.92	4.45 (0.64)
“Did you know?” - Relevant, appropriate content presented clearly and objectively	0.96	4.42 (0.66)	0.96	4.42 (0.66)
**Relevance**				
Relevant to PHC nurses	0.87	4.24 (0.70)	0.87	4.24 (0.70)
**Layout and images**				
The game’s design and appearance are appealing and appropriate, with a clear visual layout on the cards and board	0.87	4.26 (0.76)	0.91	4.47 (0.72)
**Linguistic accuracy**				
Appropriate font size and typeface, with a style suited to the target audience’s level of knowledge	0.72	3.86 (1.01)	0.95	4.76 (0.45)
**S-CVI/Ave**	**0.84**		**0.93**	

*I-CVI - Item-level Content Validity Index; SD - Standard Deviation; S-CVI/Ave - Average of the content validity indices across all items.*

In the first round, individual I-CVI values ranged from 0.62 to 1.00. The lowest scores were concentrated in the Linguistic accuracy, Content, and Layout and images dimensions. Items with indices below the minimum consensus threshold of 0.80 were revised based on the experts’ recommendations, especially those related to font size, writing style, clarity of illustrations, and presentation of the card content.

Analysis of the card content showed that the lowest I-CVI values, ranging from 0.58 to 0.77, were concentrated mainly in the Conflict management category. Content in the categories with indices below the cutoff point was refined for clarity, scientific accuracy, and depth. As a result, scores increased substantially in the second round, thereby validating all assessed items and improving the educational material’s clarity and appropriateness.

Once the content validity stage had been completed, the game’s usability was assessed with 14 PHC nurses selected according to the inclusion and exclusion criteria. Most were women (92.9%), with a mean of 2.3 years of experience (ranging from 1 to 6 years). In terms of academic qualifications, three (21.4%) held a doctoral degree, two (14.3%) held a master’s degree, and nine (64.3%) held a graduate certificate. The usability assessment scores are presented in Table 2 and shown in Figure 1.

**Table 2:** Usability assessment of the serious game by PHC nurses using the System Usability Scale (SUS), Florianópolis, Santa Catarina, Brazil, 2025

Statements	SUS Score
I think that I would like to use this game frequently.	94.6
I found the game very difficult to understand.	92.9
I thought the game was easy to use.	100.0
I think that I would need help from a person with technical knowledge to use this game.	60.7
The different types of content and activities are well integrated.	96.4
I think the game has many flaws.	98.2
I believe that people learn to use this game quickly.	98.2
I found this game cumbersome to use.	100.0
I felt confident using the game.	94.6
I needed to learn many new things before I could play LíderEnf.	89.3
**Total System Usability Scale score**	**92.0**

*SUS - System Usability Scale.*

**Figure 1:**
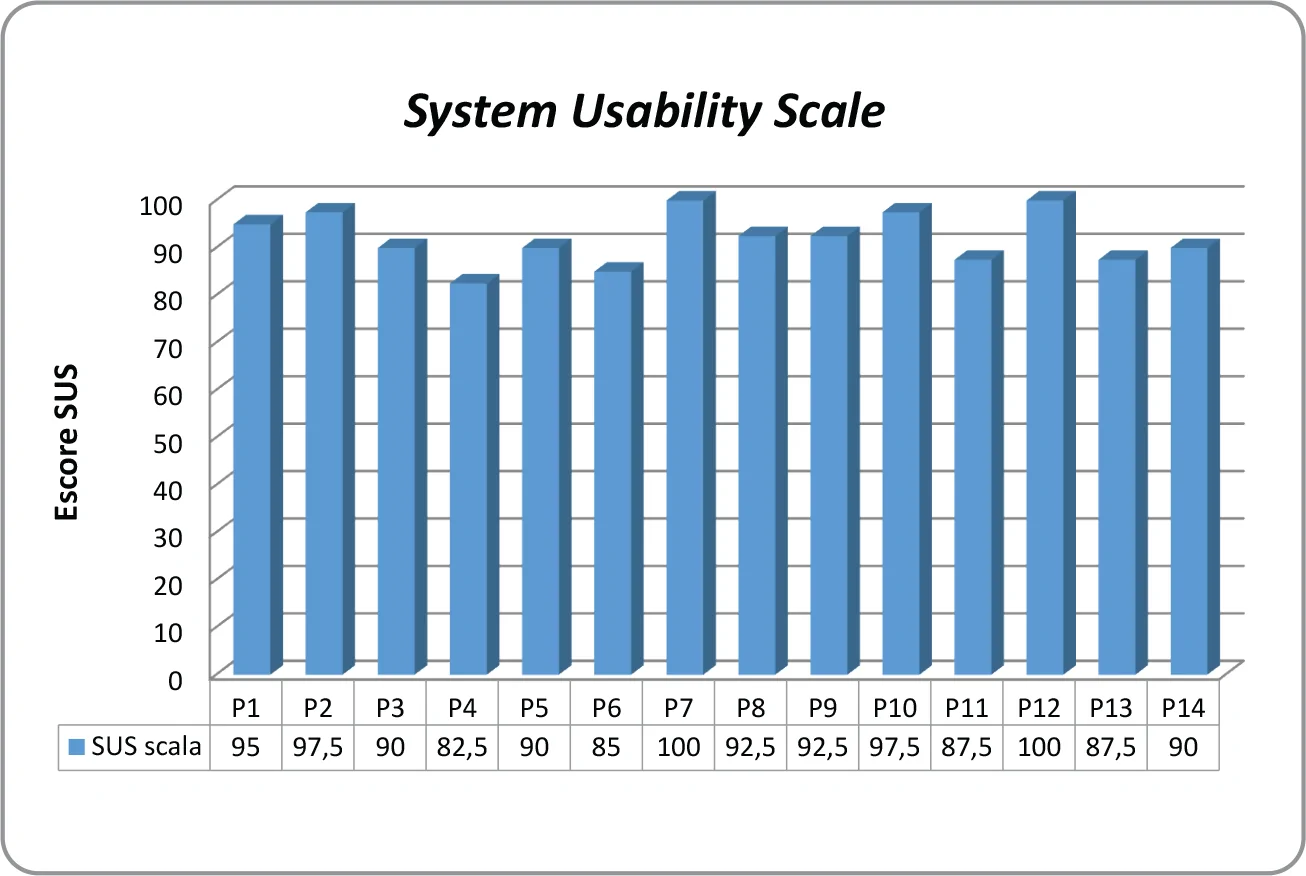
Distribution of individual System Usability Scale scores among participants (N = 14), Florianópolis, Santa Catarina, Brazil, 2025

Individual System Usability Scale scores ranged from 82.5 to 100, with an overall mean of 92.0 (SD = 5.82). Score interpretation showed that 10 participants (71.49%) rated the game in the Best Imaginable category, indicating a high level of perceived usability.

Evaluation of the educational strategy using SEGiNAS yielded a mean score of 97.9 (SD = 2.27), above the satisfactory cutoff point of 80. Item-by-item analysis showed full agreement (100%) on aspects such as encouragement of participation, usefulness for learning, suitability for content review, sparking interest in leadership, recommending the game to other professionals, and overall satisfaction with the strategy. The item related to the length of time needed for the game to support learning showed the lowest agreement (92.9%). These findings indicate strong agreement among participants regarding the serious game’s effectiveness as an educational resource.

Following these stages, the game underwent content and design refinements, including improvements to its visual organization, board layout, and card wording. The changes included increasing the font size, refining the illustrations, and revising the content and rules, making the resource clearer, more appealing, and more accessible. Figure 1 shows the final version of the LíderEnf - Primary Health Care serious game, made available to readers through the QR code shown in Figure 2. The QR code provides access to the digital version for interactive viewing. A video demonstrating the game dynamics is available on YouTube: https://www.youtube.com/watch?v=mtXHfiFr2rE

**Figure 2:**
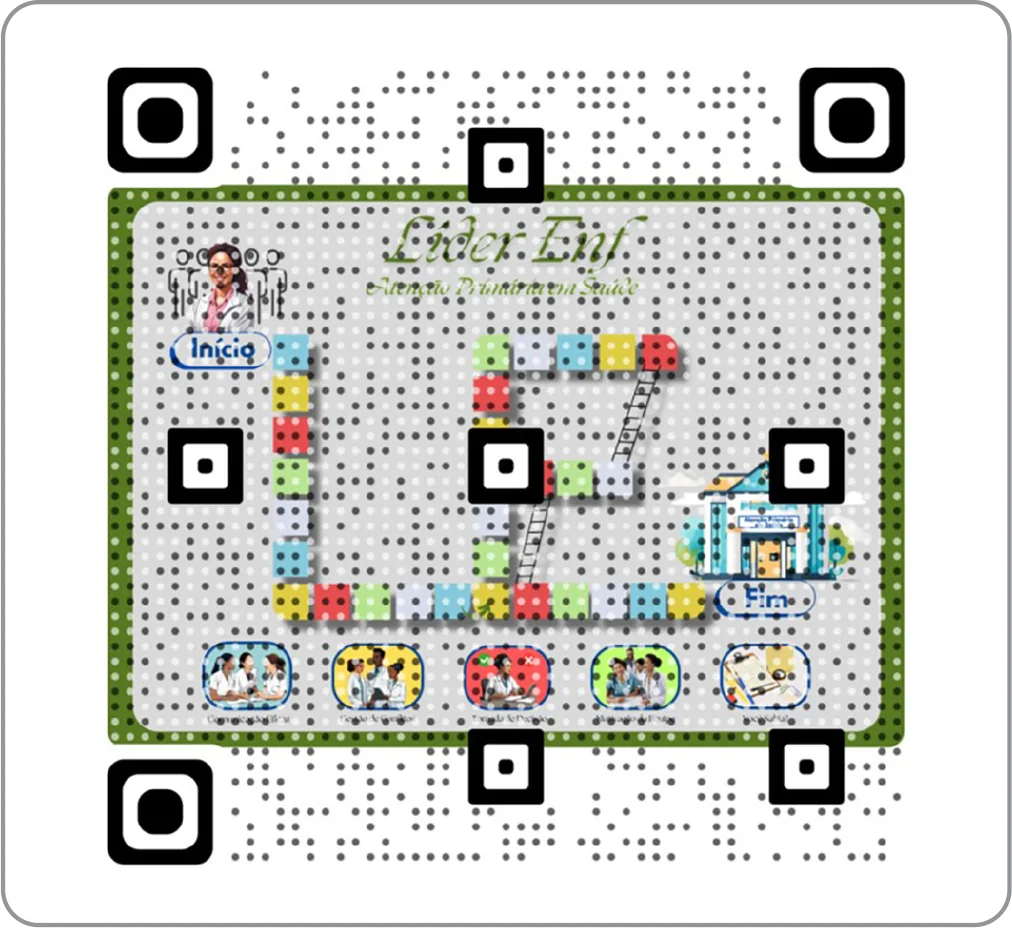
Final version of the LíderEnf - Primary Health Care serious game, with a QR code providing access to the digital version, Florianópolis, Santa Catarina, Brazil, 2025

## DISCUSSION

The development and validation of LíderEnf, intended to strengthen leadership competencies among PHC nurses, showed that this educational technology enabled participants to revisit leadership skills and reflect on their professional practice. This finding reinforces the potential of games in health professions learning and for bridging knowledge and practice in everyday work^(16,17)^. Effective leadership in PHC is directly associated with the quality of care and service responsiveness to local needs^(7)^, underscoring the relevance of strategies designed to strengthen this professional attribute.

Board games have characteristics that encourage active participation and can stimulate critical thinking. They differ from traditional approaches by incorporating competition, cooperation, and reflection, thereby expanding learning opportunities^(18)^. These resources also foster engagement, motivation, and collaborative knowledge construction in health education settings^(19)^. In addition, serious games may enhance not only technical learning but also self-management and professional self-efficacy, both of which are linked to self-leadership^(20)^.

The two-round e-Delphi process established expert consensus on the clarity, relevance, and appropriateness of the proposal. This step is essential for ensuring the legitimacy and applicability of educational technologies. Clear, representative components contribute to the material’s effectiveness, while incorporating expert feedback strengthens its scientific basis and broadens its pedagogical potential^(16)^. High validity indices have also been reported in educational health games, such as one on first aid^(9)^ and another on bioethics^(21)^, in line with the findings of this study.

The revisions made after the first round focused mainly on the card content and were accompanied by changes to the game instructions. These modifications improved clarity and organization, including a clearer definition of how the game ends. A similar process was reported in the development of the Match game^(16)^, in which experts also recommended changes to the game mechanics and instructions.

The validation of the card content, organized into thematic categories, also proved relevant to the PHC context. These dimensions align with attributes recognized in the nursing leadership literature, which describes leadership as an intrinsic professional competency involving decision-making, conflict management, effective communication, and negotiation-elements linked to preparing creative, critical, reflective, and politically aware professionals^(1,22)^. This alignment suggests that the game may support both interpersonal leadership processes and aspects of self leadership, fostering nurses’ autonomy in complex situations^(20)^. Regarding graphic design and layout, experts recommended adjustments to improve accessibility and visual clarity. Changes included larger font sizes, a revised arrangement of graphic elements, and refinements to the illustrations. Evidence suggests that the design quality of educational resources influences comprehension and facilitates user uptake^(9,23)^.

The game’s language was revised, with wording refined and instructions simplified. This review helped make the game better suited to PHC nurses’ level of knowledge. Educational technologies in health should have a simple, objective structure and use clear language tailored to users’ profiles to enhance their educational potential^(19)^.

Usability assessment is a central step in developing educational technologies in health because it makes it possible to assess, with real users, the product’s effectiveness, efficiency, and user satisfaction^(24)^. In this study, the System Usability Scale classified the game as Best Imaginable, suggesting strong acceptance in terms of ease of use, clarity, and applicability in educational settings, and supporting its feasibility for educational use. These findings are consistent with previous studies on educational technologies in health^(25,26)^. Although item-level analysis of the System Usability Scale showed agreement that the instructions were simple, the lowest scores were observed in items related to the need for initial support and the learning curve. This finding suggests that the game may be introduced more effectively when accompanied by initial guidance, as reported in recent studies on the implementation of games in health^(27)^.

The SEGiNAS findings further reinforced this positive evaluation. Participants reached consensus on items related to the resource’s usefulness for learning, encouragement of participation, and overall satisfaction. Similar results were reported in a systematic review that found a positive impact of serious games on knowledge acquisition and skills development in nursing^(28)^. These results are also consistent with studies linking leadership style in PHC teams to improved team satisfaction and motivation-factors essential to strengthening collaborative work in health teams^(7)^.

The integrated analysis of the System Usability Scale and SEGiNAS results suggests that LíderEnf has good usability and is perceived as an educational resource applicable to the PHC context. In this setting, the game helps establish leadership as a strategic axis of nursing practice by bringing together technical, relational, and organizational dimensions of care.

### Study limitations

The main limitation was the geographic concentration of the experts, who were drawn predominantly from the South and Southeast Regions of Brazil, which may have reduced the diversity of perspectives represented, despite invitations sent to professionals from other regions. Although the size of the expert panel does not, in itself, constitute a methodological limitation, the relatively small number of participants may still narrow the range of opinions captured. In addition, the study did not assess the impact of the serious game on professional practice, which limits understanding of its effectiveness in real-world PHC settings.

### Contributions to the field of nursing

The validation of LíderEnf provides the nursing field with an educational technology designed to strengthen leadership in the PHC context. By integrating theory and practice through a playful, participatory approach, the game broadens opportunities for continuing education. It also deepens critical reflection on management, communication, and decision-making processes, which are central to interprofessional teamwork and to improving the quality of care.

In public health and public policy, the proposal also aligns with the SDGs by promoting pedagogical innovation, workforce strengthening, and stronger PHC. The adoption of validated educational technologies such as LíderEnf can support continuing education policies, contribute to more integrated care practices, and strengthen institutional responsiveness to population needs.

## CONCLUSIONS

The validation process showed that the educational technology developed has a sound theoretical basis for strengthening leadership competencies among PHC nurses. Results from the content validity, usability, and educational strategy appraisal stages showed high levels of agreement among experts and users, supporting the game’s applicability and pedagogical potential. The resource proved valid, highly usable, and effective in supporting the development of leadership competencies, offering an innovative alternative to traditional educational practices.

LíderEnf proved applicable to the PHC context and may be integrated into training and continuing education processes in health care. Future studies should include different settings and assess its impact on professional practice, expanding understanding of its effectiveness in real world work contexts.

## Data Availability

The research data are not available.
