# Supplementary material for: Development and validation of a serious game for nursing leadership in Primary Health Care
Source: Rev Bras Enferm. 2026 Sep 25;79:e20250590. doi: 10.1590/0034-7167-2025-0590 (PMC13619154; doi:10.1590/0034-7167-2025-0590)
Supplement: Translation

# Translation: Liderança em enfermagem na Atenção Primária à Saúde: elaboração e validação de um jogo sério

**DOI:** 10.1590/0034-7167-2025-0590pt

**Authors:** Caren Cristina Wiles Della Méa da Fonseca, Alacoque Lorenzini Erdmann, Carla Sílvia Fernandes, Alexandre Pazetto Balsanelli, Ana Izabel Jatobá de Souza, Keyla Cristiane do Nascimento

**Affiliations:** I Universidade Federal de Santa Catarina. Florianópolis, Santa Catarina, Brasil; II Escola Superior de Saúde, Instituto Politécnico de Viana do Castelo. Viana do Castelo, Portugal; III Universidade Federal de São Paulo. São Paulo, São Paulo, Brasil

**Keywords:** Estudo de Validação, Tecnologia Educacional, Liderança, Enfermagem, Atenção Primária à, Saúde.

## Abstract

**Objetivos:**

elaborar e validar um jogo sério sobre liderança para enfermeiros da Atenção Primária à Saúde.

**Métodos:**

estudo metodológico, quantitativo e descritivo, em duas etapas: validação de conteúdo por 13 especialistas, via técnica e-Delphi; e avaliação de usabilidade por 14 enfermeiros, utilizando a *System Usability Scale* e o *Serious Educational Game in Nursing Appraisal Scale*. Os dados foram analisados por estatística descritiva.

**Resultados:**

o Índice de Validade de Conteúdo global foi 0,93. A usabilidade obteve média de 92 pontos; e a avaliação como estratégia educativa, 97,9 pontos. A versão final do *jogo sério* constituiu-se de tabuleiro com layout aprimorado, 75 cartas revisadas e regras ajustadas, resultando em um recurso aplicável à prática profissional.

**Conclusões:**

o *jogo sério* mostrou-se uma tecnologia educacional válida, de alta usabilidade e eficaz para desenvolver competências de liderança entre enfermeiros da Atenção Primária, configurando-se como alternativa inovadora às práticas educativas tradicionais.

## INTRODUÇÃO

A liderança é considerada uma competência essencial na prática da enfermagem, exercendo impacto direto na qualidade da assistência aos pacientes, fortalecimento dos vínculos familiares, coordenação das equipes e gerenciamento dos serviços de saúde^(1)^. Investir no desenvolvimento da liderança e qualificação dos enfermeiros constitui estratégia central para fortalecer os sistemas de saúde e alcançar os Objetivos de Desenvolvimento Sustentável (ODS) da Agenda 2030^(2,3)^.

Na Atenção Primária à Saúde (APS), esse desafio assume maior complexidade, pois o exercício da liderança pelo enfermeiro requer não apenas habilidades técnicas, mas também competências relacionais e gerenciais, necessárias para atuar em ambientes dinâmicos e complexos^(4)^. Esse processo demanda reflexão contínua, postura ética, capacidade de construir vínculos de confiança com pacientes e equipes multiprofissionais e de promover efetivamente o trabalho colaborativo^(1)^.

Entre as competências relacionadas à liderança dos enfermeiros, destacam-se a tomada de decisão, a gestão de conflitos, a comunicação assertiva, o pensamento crítico e a empatia. Tais competências melhoram a prática administrativa, assistencial, educacional e de pesquisa, devendo ser incentivadas ainda durante a formação, para que os futuros enfermeiros estejam mais seguros e aptos a exercê-las^(5)^. Estudos recentes mostram, entretanto, que ainda existem lacunas formativas e dificuldades na consolidação dessas competências, tanto na formação inicial quanto no desenvolvimento profissional contínuo^(5,6)^.

Evidências no âmbito da APS apontam desafios relacionados às competências gerenciais e de liderança dos enfermeiros, reforçando a necessidade de programas estruturados de educação permanente e iniciativas voltadas ao desenvolvimento de lideranças^(7)^. Investir em processos educativos alinhados às demandas reais dos serviços é essencial para transformar práticas de cuidado, aumentar a resolutividade das ações e qualificar a atenção à saúde^(6)^.

Nesse cenário, as tecnologias educacionais vêm se consolidando como alternativas eficazes na formação e no desenvolvimento profissional em saúde. Os jogos sérios, uma categoria de jogos educativos, destacam-se por estimular a participação, a motivação e a construção coletiva do conhecimento, integrando elementos de competição saudável, reflexão crítica e engajamento criativo^(8)^. Em especial, os jogos de tabuleiro apresentam-se como um recurso pedagógico ativo, dinâmico e inovador, capaz de traduzir conteúdos complexos em experiências acessíveis e participativas, favorecendo a aprendizagem significativa e a aplicação prática dos saberes adquiridos^(9,10)^.

Apesar da ampliação do uso dessas tecnologias, ainda são poucos os estudos que exploram recursos voltados à liderança de enfermeiros na APS. Essa lacuna limita o avanço científico e restringe a incorporação de estratégias pedagógicas inovadoras no cotidiano dos serviços de saúde. Nesse sentido, a inserção de jogos educativos apresenta-se como alternativa metodológica capaz de favorecer a construção do conhecimento de forma contextualizada e aplicável a diferentes situações.

## OBJETIVOS

Elaborar e validar um jogo sério sobre liderança para enfermeiros da Atenção Primária à Saúde.

## MÉTODOS

### Aspectos éticos

O estudo atendeu às normas éticas nacionais e internacionais para pesquisas com seres humanos. Foi aprovado pelo Comitê de Ética em Pesquisa da Universidade Federal de Santa Catarina (UFSC), sob CAAE nº 78961724.0.0000.0121 e parecer nº 6.818.857. As imagens utilizadas no jogo foram obtidas de banco de imagens com licença isenta de royalties, respeitando os direitos autorais da plataforma Canva^®^.

### Desenho, período e local do estudo

Trata-se de um estudo metodológico, de abordagem quantitativa e descritiva, norteado pelas recomendações do *Guidelines for reporting reliability and agreement studies* (GRRAS), da EQUATOR Network, adaptadas para pesquisas de validação de tecnologias educacionais. Foi conduzido entre fevereiro de 2024 e abril de 2025, em uma capital da Região Sul do Brasil. O desenvolvimento do jogo seguiu o modelo Hexa-Tau, direcionado à construção de jogos de tabuleiro educacionais^(11)^. O percurso metodológico compreendeu três etapas inter-relacionadas: 1) desenvolvimento e concepção do jogo sério, 2) validação de conteúdo por especialistas e 3) avaliação da usabilidade e aplicabilidade com enfermeiros da APS.

### População; critérios de inclusão e exclusão

Na etapa de validação de conteúdo, os especialistas foram selecionados por amostragem não probabilística de conveniência por meio da Plataforma Lattes do Conselho Nacional de Desenvolvimento Científico e Tecnológico (CNPq), considerando a expertise na temática “liderança”. Estabeleceram-se como critérios de inclusão: ser enfermeiro, ter experiência na área de liderança em enfermagem na APS e atuar há pelo menos um ano nesse nível de atenção. O convite foi enviado a 32 profissionais, via e-mail, incluindo link para formulário eletrônico. Após o consentimento em participar, o acesso ao instrumento era liberado; em caso de recusa, o processo era encerrado. Foram excluídos os que não retornaram o instrumento no prazo estabelecido.

Para a avaliação de usabilidade, foram convidados 29 enfermeiros atuantes na APS de um município do Sul do Brasil. Os critérios de inclusão foram: ser enfermeiro da APS e exercer a função há pelo menos um ano. Foram excluídos os profissionais afastados por férias ou licença durante o período da coleta de dados. A participação na pesquisa não interferiu nas atividades profissionais dos enfermeiros, uma vez que a coleta foi conduzida fora do contexto assistencial. Ressalta-se que todos os enfermeiros foram convidados a participar das atividades do jogo, independentemente de sua decisão quanto à pesquisa, visto que se tratava de uma atividade de caráter lúdico e inclusivo. Os questionários foram aplicados aos indivíduos que aceitaram participar do estudo, em conformidade com o Termo de Consentimento Livre e Esclarecido.

### Protocolo do estudo

#### 1ª etapa - Desenvolvimento do jogo sério

Na primeira etapa, realizou-se a concepção do jogo, desenvolvido com base no modelo Hexa-Tau^(11)^. Optou-se pela elaboração de um jogo analógico, em formato de tabuleiro, por sua acessibilidade, facilidade de replicação e potencial de promover interação em atividades educativas presenciais. O jogo, intitulado “LíderEnf - Atenção Primária à Saúde”, foi estruturado em formato retangular (42 × 30 cm), composto por 30 casas coloridas, cada uma representando diferentes habilidades de liderança. O percurso do tabuleiro foi concebido de modo a formar graficamente as letras “L” e “E”, iniciais de “Liderança” e “Enfermagem”, em alusão ao nome do jogo. As casas, de cores variadas (vermelho, azul, verde, amarelo e branco), foram dispostas de forma contínua e interligadas por escadas que simulam avanços estratégicos, e as cores foram utilizadas para identificar as categorias temáticas. O conjunto inclui quatro peões, um dado de seis faces, um manual de regras e 75 cartas com perguntas e afirmações, organizadas em categorias temáticas relacionadas às competências de liderança em enfermagem: comunicação eficaz, tomada de decisão, gestão de conflitos, motivação da equipe e cartas informativas (“Você sabia?”)^(11)^.

#### 2ª etapa - Validação de conteúdo

A avaliação considerou clareza, relevância e organização, utilizando a técnica e-Delphi^(12)^. O questionário, estruturado no *Google Forms* ^®^, incluiu a caracterização dos especialistas (idade, sexo, formação e outros aspectos) e 32 itens distribuídos em seis dimensões: Objetividade, Conteúdo, Relevância, Organização, Layout/imagens e Precisão linguística. Cada item foi avaliado em escala Likert de cinco pontos (de 1 = Discordo totalmente até 5 = Concordo totalmente)^(13)^. Itens com pontuação inferior a 4 foram acompanhados de comentários e sugestões. O processo foi conduzido em duas rodadas, até atingir consenso mínimo de 80% no Índice de Validade de Conteúdo (IVC)^(13)^.

#### 3ª etapa - Avaliação de usabilidade

A avaliação da usabilidade foi realizada presencialmente com os enfermeiros em seis encontros nas unidades de saúde da APS, organizados conforme a disponibilidade dos participantes. Como se tratava de um jogo de tabuleiro, cada sessão contou com, no mínimo, dois participantes para garantir a dinâmica da atividade. As sessões tiveram duração média de uma hora, abrangendo a realização de uma rodada completa do jogo e a aplicação dos instrumentos de avaliação. Foram utilizados dois instrumentos: o *System Usability Scale* (SUS), versão traduzida e validada para o português, composta por 10 itens^(14)^; e o *Serious Educational Game in Nursing Appraisal Scale* (SEGiNAS), contendo 20 afirmações distribuídas em três dimensões (Envolvimento e Eficácia no Ensino, Impacto da Aprendizagem e Aplicação Prática e Relevância e Clareza do Conteúdo)^(15)^. Ambos os instrumentos foram respondidos em escala Likert de cinco pontos, variando de “Discordo totalmente” a “Concordo totalmente”.

### Análise dos dados e estatística

Os dados foram organizados em planilhas do Microsoft Excel 365^*®*^ e analisados por meio de estatística descritiva, utilizando frequências absolutas e relativas para a caracterização dos participantes. O IVC foi calculado com base na soma das respostas “4” e “5”, dividida pelo total de respostas para cada item. Adotou-se como aceitável o valor mínimo de 0,80 (80%). Itens com valor inferior foram ajustados conforme as sugestões dos juízes e reavaliados em uma segunda rodada, enquanto os que já haviam alcançado consenso na primeira rodada foram considerados válidos. Para o *System Usability Scale*, a pontuação foi calculada conforme recomendações originais: nos itens ímpares, subtraiu-se 1 do valor atribuído; nos itens pares, o valor atribuído foi subtraído de 5. A soma dos valores foi multiplicada por 2,5, resultando em um escore entre 0 e 100. Os escores foram classificados em valores médios do *System Usability Scale* associados a adjetivos qualitativos: 12,5 (Pior imaginável), 20,3 (Muito ruim), 35,7 (Ruim), 50,9 (Regular), 71,4 (Bom), 85,5 (Excelente) e 90,9 (Melhor imaginável)^(14)^. O SEGiNAS foi analisado por somatória simples, sendo considerado satisfatório o escore mínimo de 80 pontos (80% de concordância).

## RESULTADOS

Participaram da validação 13 enfermeiros especialistas com experiência em liderança, sendo oito (61,5%) na faixa etária entre 40 e 50 anos, com predomínio do sexo feminino (84,6%). O tempo médio de formação foi de 21,6 anos. Quanto à localização geográfica, nove (69,2%) eram provenientes da Região Sul e quatro (30,8%) da Região Sudeste do Brasil. No tocante à titulação acadêmica, cinco (38,5%) tinham pós-doutorado; quatro (30,8%), doutorado; e quatro (30,8%), mestrado. Quanto à atividade profissional, seis (46,2%) atuavam como docentes e sete (53,8%) como enfermeiros da APS.

Na técnica e-Delphi, os itens de validação de conteúdo foram avaliados individualmente pelos especialistas, com base na frequência de concordância e cálculo do IVC. Os resultados das duas rodadas estão consolidados na Tabela 1, que apresenta os valores de IVC e as médias atribuídas em cada dimensão analisada.

**Tabela 1:** Índice de Validade de Conteúdo e médias dos itens avaliados nas duas rodadas da técnica e-Delphi (n = 13; n = 9), Florianópolis, Santa Catarina, Brasil, 2025

Dimensões de validação	e-Delphi 1 (n = 13)		e-Delphi 2 (n = 09)	
	IVC-I	Média (DP)	IVC-I	Média (DP)
**Objetividade**				
Objetivos claros, coerentes com a temática de liderança	0,90	4,58 (0,66)	0,90	4,58 (0,66)
**Organização**				
Organização e regras do jogo são claras, objetivas, coerentes e de fácil entendimento	0,87	4,28 (0,91)	0,88	4,36 (0,83)
**Conteúdo**				
Comunicação eficaz - Conteúdo relevante, adequado e apresentado de forma clara e objetiva	0,79	4,08 (1,03)	0,98	4,57 (0,57)
Tomada de decisão - Conteúdo relevante, adequado e apresentado de forma clara e objetiva	0,83	4,19 (1,00)	0,91	4,34 (0,84)
Gestão de conflitos - Conteúdo relevante, adequado e apresentado de forma clara e objetiva	0,78	4,10 (1,08)	0,97	4,50 (0,62)
Motivação da equipe - Conteúdo relevante, adequado e apresentado de forma clara e objetiva	0,84	4,10 (1,02)	0,92	4,45 (0,64)
Você sabia - Conteúdo relevante, adequado e apresentado de forma clara e objetiva	0,96	4,42 (0,66)	0,96	4,42 (0,66)
**Relevância**				
Relevante para enfermeiros da APS	0,87	4,24 (0,70)	0,87	4,24 (0,70)
**Layout e imagens**				
O design e a aparência do jogo são atrativos e adequados, com interface clara nas cartas e no tabuleiro	0,87	4,26 (0,76)	0,91	4,47 (0,72)
**Precisão linguística**				
Apresenta tamanho e fonte adequados, estilo compatível com o conhecimento do público-alvo	0,72	3,86 (1,01)	0,95	4,76 (0,45)
**S-CVI/Ave**	**0,84**		**0,93**	

*IVC-I - Índice de Validade de Conteúdo dos itens individuais; DP - Desvio-padrão; S-CVI/Ave - Média dos índices de validação de conteúdo para todos os índices obtidos.*

Na primeira rodada, os valores individuais do IVC variaram entre 0,62 e 1,00. As menores pontuações concentraram-se nas dimensões “Precisão linguística”, “Conteúdo” e “Layout e imagens”. Índices abaixo do consenso mínimo de 0,80 foram ajustados conforme as recomendações dos especialistas, especialmente aqueles relacionados ao tamanho da fonte, estilo textual, clareza das ilustrações e apresentação de conteúdo das cartas.

A análise do conteúdo das cartas evidenciou que os menores IVCs, variando entre 0,58 e 0,77, concentravam-se principalmente na categoria temática “Gestão de conflitos”. Os conteúdos das categorias com índices abaixo do ponto de corte foram aprimorados em termos de clareza, adequação científica e profundidade do conteúdo. Como resultado, houve elevação significativa dos escores na segunda rodada, garantindo a validação de todos os itens avaliados e maior clareza e adequação do material educativo.

Concluída a etapa de validação de conteúdo, procedeu-se à avaliação de usabilidade do jogo, com a participação de 14 enfermeiros da APS selecionados com base nos critérios de inclusão e exclusão. A maioria era do sexo feminino (92,9%), com tempo médio de experiência de 2,3 anos (variando de 1 a 6 anos). Quanto à titulação acadêmica, três (21,4%) tinham doutorado; dois (14,3%), mestrado; e nove (64,3%), especialização. Os escores obtidos na avaliação de usabilidade estão apresentados na Tabela 2 e ilustrados na Figura 1.

**Tabela 2:** Avaliação de usabilidade do jogo sério por enfermeiros da APS, conforme o *System Usability Scale* (SUS), Florianópolis, Santa Catarina, Brasil, 2025

Afirmações	Escore SUS
Eu gostaria de usar esse jogo com frequência.	94,6
Eu achei o jogo muito difícil de entender.	92,9
Eu achei o jogo fácil de usar.	100,0
Eu acho que precisa de ajuda de uma pessoa com conhecimentos técnicos para usar esse jogo.	60,7
A variação de conteúdos e atividades estão bem integrados.	96,4
Eu acho que o jogo apresenta muitas falhas.	98,2
Eu acredito que as pessoas aprendem a usar esse jogo rapidamente.	98,2
Eu achei esse jogo complicado de usar.	100,0
Eu me senti confiante ao usar o jogo.	94,6
Eu precisei aprender várias coisas novas antes de conseguir jogar o LíderEnf.	89,3
**Escore total *System Usability Scale***	**92,0**

*SUS - System Usability Scale.*

**Figura 1:** Distribuição dos escores individuais do *System Usability Scale* entre os participantes (N = 14), Florianópolis, Santa Catarina, Brasil, 2025

Os escores da *System Usability Scale* individuais dos participantes variaram entre 82,5 e 100 pontos, com média geral de 92,0 (DP = 5,82). A interpretação dos escores demonstrou que 10 participantes (71,49%) atribuíram pontuações situadas na categoria “Melhor imaginável”, evidenciando elevado nível de usabilidade percebida.

A avaliação da estratégia educativa por meio do SEGiNAS resultou em escore médio de 97,9 (DP = 2,27), acima do ponto de corte de 80 estabelecido como satisfatório. Na análise item a item, verificou-se concordância total (100%) em aspectos como incentivo à participação, utilidade para a aprendizagem, adequação para revisão de conteúdos, despertar de interesse pela liderança, recomendação a outros profissionais e satisfação geral com a estratégia. O item referente à duração necessária do jogo para permitir a aprendizagem apresentou a menor concordância (92,9%). Tais resultados evidenciam elevado nível de concordância entre os participantes em relação à efetividade do jogo sério enquanto recurso formativo.

Após essas etapas, o jogo passou por ajustes de conteúdo e design, com melhorias na organização visual, no layout do tabuleiro e na redação das cartas. As modificações incluíram o aumento do tamanho das fontes, a adequação das ilustrações e a revisão dos conteúdos e das regras, tornando o recurso mais claro, atrativo e acessível. A Figura 1 apresenta a versão final do jogo sério LíderEnf - Atenção Primária à Saúde, disponibilizada aos leitores via *QR Code* (Figura 2). O *QR Code* dá acesso à versão digital para visualização interativa. O vídeo sobre a dinâmica do jogo está disponível no YouTube: https://www.youtube.com/watch?v=mtXHfiFr2rE.

**Figura 2:** Versão final do jogo sério LíderEnf - Atenção Primária à Saúde, com *QR code* para acesso à versão digital, Florianópolis, Santa Catarina, Brasil, 2025

## DISCUSSÃO

A elaboração e validação do LíderEnf, voltado ao fortalecimento de competências de liderança de enfermeiros da APS, mostraram que essa tecnologia educacional permitiu aos participantes revisitarem habilidades de liderança e refletir sobre suas práticas profissionais. Tal resultado reforça o potencial dos jogos na aprendizagem em saúde e na integração entre conhecimento e prática no cotidiano de trabalho^(16,17)^. A liderança efetiva no âmbito da APS está diretamente associada à qualidade do cuidado e à capacidade de resposta dos serviços às necessidades locais^(7)^, o que reforça a pertinência de estratégias voltadas ao fortalecimento desse atributo profissional.

Jogos de tabuleiro apresentam características que favorecem a participação ativa e podem estimular o pensamento crítico. Diferenciam-se das abordagens tradicionais ao incorporar elementos de competição, cooperação e reflexão, que ampliam as possibilidades de aprendizagem^(18)^. Esses recursos também propiciam o engajamento, a motivação e a construção do conhecimento em contextos educacionais em saúde^(19)^. Além disso, os jogos sérios podem potencializar não apenas a aprendizagem técnica, mas também processos de autogestão e autoeficácia profissional, aspectos vinculados ao conceito de *self-leadership* ^(20)^.

A validação de conteúdo em duas rodadas e-Delphi evidenciou consenso entre os especialistas quanto à clareza, relevância e adequação da proposta. Esse processo é uma etapa necessária para assegurar a legitimidade e a aplicabilidade de tecnologias educacionais. A clareza e representatividade dos componentes colaboram para a eficácia do material, enquanto a incorporação das contribuições fortalece a base científica e amplia seu potencial pedagógico^(16)^. Índices elevados de validade também foram observados em jogos educativos em saúde, como em um jogo sobre primeiros socorros^(9)^ e em outro sobre bioética^(21)^, corroborados pelos resultados deste estudo.

Os ajustes realizados após a primeira rodada concentraram-se principalmente no conteúdo das cartas e foram acompanhados de modificações nas instruções do jogo. As mudanças implementadas contribuíram para maior clareza e organização, incluindo uma definição mais objetiva do encerramento da partida. Processo semelhante foi relatado no desenvolvimento do jogo Match^(16)^, no qual especialistas também recomendaram alterações relacionadas à mecânica e às instruções.

A validação do conteúdo das cartas, organizadas em categorias temáticas, mostrou-se pertinente ao contexto da APS. Essas dimensões estão alinhadas com atributos reconhecidos na literatura sobre liderança em enfermagem, descrita como competência intrínseca ao exercício profissional, envolvendo tomada de decisões, gerenciamento de conflitos, comunicação efetiva e negociação - aspectos vinculados à formação de profissionais criativos, críticos, reflexivos e politizados^(1,22)^. Esse alinhamento sugere que o jogo pode apoiar tanto processos de liderança interpessoal quanto aspectos de autoliderança, favorecendo a autonomia do enfermeiro em situações complexas^(20)^. Em relação ao design gráfico e ao layout, os especialistas recomendaram ajustes voltados à acessibilidade e à clareza visual. Foram incorporadas alterações no tamanho da fonte, na disposição dos elementos gráficos e na adequação das ilustrações. Evidências sugerem que a qualidade do design de recursos educacionais influencia a compreensão das informações e favorece a adesão dos usuários^(9,23)^.

A linguagem utilizada no jogo foi revisada, com ajustes na redação e simplificação das instruções. Essa etapa de revisão contribuiu para que o jogo se tornasse mais adequado ao nível de conhecimento dos enfermeiros da APS. Tecnologias educacionais em saúde devem apresentar estrutura simples, objetiva e empregar linguagem clara e adaptada ao perfil dos usuários, para ampliar seu potencial formativo^(19)^.

A avaliação de usabilidade constitui etapa central no desenvolvimento de tecnologias educacionais em saúde, pois permite verificar, com usuários reais, a efetividade e a eficiência do produto, bem como a satisfação desses usuários^(24)^. No presente estudo, a *System Usability Scale* classificou o jogo como “Melhor imaginável”, sugerindo boa aceitação quanto à sua facilidade de uso, clareza e aplicabilidade em contextos formativos e demonstrando sua viabilidade para práticas educativas. Esses achados são consistentes com experiências anteriores no desenvolvimento de tecnologias educacionais em saúde^(25,26)^. Embora a análise dos itens da *System Usability Scale* tenha mostrado concordância quanto à simplicidade das instruções, os menores escores foram observados na necessidade de apoio inicial e na curva de aprendizagem. Isso sugere que a introdução do jogo poderia ser mais eficaz com orientações iniciais, conforme demonstram estudos recentes sobre implementação de jogos em saúde^(27)^.

A aplicação do SEGiNAS reforçou a avaliação positiva. Houve consenso entre os participantes em itens relacionados à utilidade do recurso para aprendizagem, incentivo à participação e satisfação geral. Resultados semelhantes foram descritos em revisão sistemática, que identificou impacto positivo dos jogos sérios na aquisição de conhecimento e desenvolvimento de habilidades em enfermagem^(28)^. Esses resultados também se alinham a estudos que associam o estilo de liderança em equipes de APS à melhoria da satisfação e motivação da equipe - fatores essenciais para fortalecer o trabalho colaborativo nas equipes de saúde^(7)^.

A análise integrada dos resultados obtidos com a *System Usability Scale* e o SEGiNAS indica que o LíderEnf apresenta boa usabilidade e é percebido como recurso pedagógico aplicável à realidade da APS. Nesse cenário, o jogo contribui para consolidar a liderança enquanto eixo estratégico da prática de enfermagem, articulando dimensões técnicas, relacionais e organizacionais no cuidado.

### Limitações do estudo

A principal limitação refere-se à concentração geográfica dos especialistas, provenientes majoritariamente das Regiões Sul e Sudeste do Brasil, o que pode ter reduzido a diversidade de perspectivas contempladas, apesar dos convites encaminhados a profissionais de outras regiões. Ainda que o tamanho do painel de especialistas não configure limitação metodológica, o número reduzido de participantes pode ser entendido como restrição à variedade de opiniões. Além disso, não foi realizada a avaliação do impacto do jogo sério na prática profissional, o que limita a compreensão de sua efetividade em contextos reais da APS.

### Contribuições para a área da enfermagem

A validação do LíderEnf oferece à enfermagem uma tecnologia educacional voltada ao fortalecimento da liderança no contexto da APS. O jogo amplia as possibilidades de formação continuada ao integrar teoria e prática em uma abordagem lúdica e participativa. Assim, aprofunda se a reflexão crítica sobre processos de gestão, comunicação e tomada de decisão, que são aspectos centrais para o trabalho em equipes multiprofissionais e para a qualificação do cuidado.

No campo da saúde coletiva e das políticas públicas, a proposta alinha-se com os ODS ao promover inovação pedagógica, qualificação da força de trabalho e fortalecimento da APS. A adoção de tecnologias educacionais validadas, como o LíderEnf, pode apoiar políticas de educação permanente, contribuir para práticas assistenciais mais integradas e reforçar a capacidade institucional de resposta às necessidades da população.

## CONCLUSÕES

A validação demonstrou que a tecnologia educacional desenvolvida apresenta adequação teórica para o fortalecimento das competências de liderança entre enfermeiros da APS. Os resultados obtidos nas etapas de validação de conteúdo, usabilidade e avaliação da estratégia educativa indicaram elevados índices de concordância entre especialistas e usuários, evidenciando a aplicabilidade e o potencial pedagógico do jogo. O recurso demonstrou-se válido, altamente usável e efetivo no fortalecimento das competências de liderança, constituindo uma alternativa inovadora às práticas educativas tradicionais.

O LíderEnf mostrou-se aplicável ao contexto da APS, com possibilidade de integração a processos de formação e educação permanente em saúde. Recomenda-se que futuras investigações incluam diferentes cenários e avaliem seu impacto na prática profissional, ampliando a compreensão sobre sua efetividade em contextos reais de trabalho.

## Data Availability

Os dados de pesquisa não estão disponíveis.
